# Terrain-Informed UAV Path Planning for Mountain Search: A Slope-Based Probabilistic Approach

**DOI:** 10.3390/s26010062

**Published:** 2025-12-21

**Authors:** Xi Wang, Xing Wang, Pengliang Zhao, Weihua Tan, Hongqiang Zhang, Lihuang Chen, Longhua Zhou

**Affiliations:** 1 The School of Information and Electrical Engineering, Hunan University of Science and Technology, Xiangtan 411100, China; 2 Sanya Institute, Hunan University of Science and Technology, Sanya 572024, China

**Keywords:** UAV, path planning, dynamic target search, slope information, probability map

## Abstract

To enhance the efficiency of locating dynamic missing persons in complex mountain terrain, this study introduces an innovative Slope Probability Search (SPS) algorithm based on a modified A* framework. The algorithm’s core is a dynamic global probability map, constructed by linking terrain slope to the behavioral tendencies of missing persons. This fundamentally shifts the unmanned aerial vehicle (UAV) search paradigm from conventional coverage patterns to intelligent, guided exploration. To ensure a realistic evaluation, we designed three representative dynamic models for the missing persons: Terrain Constrained, Path Following, and Random Walk. The SPS algorithm, through its unique heuristic function, achieves an optimal balance between exploiting high probability areas and exploring new regions to maximize search efficiency. Simulation experiments using real-world geographic data demonstrated that even under severe constraints of limited search duration and sensor range, the algorithm achieved a success rate of 88.9% achieving an average search time substantially lower than that of conventional methods. This research provides a solid theoretical basis and a practical algorithmic framework for developing next generation intelligent search and rescue systems.

## 1. Introduction

Mountainous regions, with their complex topography and volatile climate, are susceptible to natural disasters and outdoor safety incidents [1,2]. Consequently, search and rescue operations for missing persons in these environments face considerable challenges. For instance, following the 6.8 magnitude earthquake in Luding County, China, in 2022, massive landslides paralyzed the transportation network, severely impeding the prompt deployment of ground rescue teams. The success of any search and rescue (SAR) mission depends on the rapid and accurate localization of missing persons. However, traditional ground methods often consume significant time and labor, expose rescuers to hazards, and fail to meet the efficiency requirements of the critical rescue time window.

The rapid proliferation of UAVs has made them a transformative technology in numerous civil sectors [3,4,5,6]. Their role has been particularly pivotal in mountainous emergency SAR, where they are valued for high maneuverability, rapid deployment, and the ability to operate in hazardous environments [7,8,9,10]. Current research on UAV assisted SAR primarily focuses on optimizing missing person detection algorithms, coordinating multiple UAV formations, and planning search paths. For example, Martinez-Alpiste et al. proposed a real time human detection system to enhance the missing person detection capabilities of UAVs in wilderness areas [11], while Tao Xiong et al. developed an organized search method for UAV path planning [12]. Furthermore, a key challenge in enhancing SAR efficiency is the efficient deployment of UAV swarms to maximize search coverage. To this end, some studies have introduced innovative deployment algorithms, such as models based on molecular force fields, to optimize the swarm’s coverage performance [13].

However, existing SAR strategies exhibit significant limitations when applied to mountain environments. Most rely on conventional planar coverage patterns, where the search path is dictated by the size and shape of the search area. For standard rectangular zones, methods like parallel sweeps [14,15], spiral patterns [16], or random searches are common. These methods generate exhaustive paths to survey the entire area, a strategy that is effective when information about the map and missing person is unknown. In mountainous scenarios where topographical data is often available, such blanket coverage strategies are inefficient. These methods do not incorporate spatial information about the missing person’s probable location, leading to wasted resources as UAVs are directed to unlikely search areas. Furthermore, these methods are ill suited for dynamic search tasks, as they typically assume missing persons are stationary and randomly distributed.

To overcome these shortcomings, some studies have integrated geographic information into path planning. Lanjun Liu et al. used qualitative prior information to extract high probability curvilinear segments to guide UAV target detection in nonlinear regions [17]. Javier Muñoz modeled target probability distributions by fitting the positions of marine particles to optimize coverage patterns [18], and Zhonghua Hong et al. improved the efficiency of long distance path planning by incorporating terrain data [19]. Other approaches, such as Voronoi diagrams combined with Eppstein’s k best paths algorithm [20], A* [21,22], and probabilistic roadmaps [23,24], have also been explored. Nevertheless, the primary focus of these methods remains on obstacle avoidance rather than intelligent target searching.

The SAR problem is fundamentally different from traditional path planning. A conventional path planning problem seeks an optimal route between a known start and end point, navigating a known distribution of obstacles. When a missing person’s location is known, most SAR research treats it as a standard path planning problem. In actual SAR operations, however, the missing person’s location is unknown. This necessitates a dynamic search strategy that generates and adapts path in real time throughout the operation.

The behavioral patterns of missing persons have a significant impact on the selection of a search strategy. Jan-Hendrik Ewers et al. utilized psychological priors to create location probability maps of the search area to define optimal search paths [25]. In mountainous terrain, a stranded person’s travel speed, choice of direction, and resting points are intimately linked to topography, psychological state, and physical exhaustion [26,27,28]. Slope, in particular, is a critical factor influencing movement. Research indicates that average travel speed decreases significantly on slopes exceeding 10°, and each percentage point increase in slope can deter a person from choosing that path by approximately 10% [29]. To conserve energy, individuals tend to follow gentle slopes or rest at the base of inclines or on terrain benches. Failing to account for these behavioral tendencies in UAV path design leads to inefficient strategies, such as excessive area coverage and redundant flight paths, which waste the critical time available for rescue.

To address the aforementioned challenges, this paper proposes a novel UAV search strategy founded on mountain slope information. The core idea is to transform readily available terrain slope data into a dynamic prior probability map of the missing person’s location, which in turn intelligently guides the UAV’s search path. The primary contributions of this paper are as follows:

1. Slope-Based Probability Model: We introduce a model that correlates terrain slope with the known behavioral tendencies of missing persons to construct a quantified, global initial probability map. This map is dynamically updated to reflect the real time progress of the search, providing a continuously evolving information layer to guide path planning.
2. Three Representative Missing Person Motion Models: We establish three distinct dynamic motion models to characterize the potential movement patterns of missing persons during an actual SAR operation.
3. An Iterative UAV Search Algorithm: We design an algorithm that generates the UAV’s search trajectory by employing a heuristic function. This function integrates the slope probabilities, an exploration reward, and historical search information to adapt to dynamic missing persons with unknown locations. The objective is to maximize the probability of mission success while minimizing search time.

This paper is organized as follows. Section 2 elaborates on the problem formulation, the proposed models, and the dynamic probability mechanism coupled with slope. Section 3 introduces the iterative, terrain-informed UAV search algorithm. In Section 4, we compare the search efficiency of our proposed algorithm against traditional coverage methods. Finally, Section 5 and Section 6 present the discussion and conclusion, respectively.

## 2. Problem Description

A typical mountain search and rescue operation commences upon receiving an alert, where personnel first gather information to profile the missing person and establish their Last Known Point (LKP). However, this initial data is often incomplete, making the LKP highly uncertain, and a UAV’s rapid deployment capability means the search often begins while the missing person is still mobile. To model this complex scenario, this study considers a search operation where only the region’s topographical map is known beforehand. The environment is defined as a square geographical area with elevation data, within which a single UAV is deployed to find a missing person whose location is unknown, all within a fixed time frame. The UAV’s sole objective is to precisely locate the missing person, as the subsequent extraction is delegated to ground teams. Therefore, the goal of this research is to develop a search strategy that maximizes the probability of locating the missing person while minimizing the required search time.

### 2.1. Search Area

We define the search area as a bounded, 3D continuous space whose horizontal projection is a square region, denoted by $S_{xy}=[0,L]\times [0,L]$. This region is formally defined in the Cartesian coordinate system, where L represents the side length of the square. The topography within this area is described by a continuous height function, H(x,y), which assigns an elevation to each point (x,y). The complete search space, S, is therefore the continuous surface formed by these ground points, as defined in the subsequent equation.

(1) $$S=\{(x,y,z)|(x,y)\in S_{xy},z=H(x,y)\}$$

### 2.2. Missing Person

For modeling purposes, this study defines a missing person as either an individual or a group. A group is simplified and treated as a single missing person point source, meaning all members are assumed to occupy the same location and move in unison.

A single search mission may involve locating one or more independent missing persons within the same operational area. Therefore, we define the set of search missing persons as $M=\{MP_{1},MP_{2},\ldots ,MP_{N}\}$, where N ≥ 1 is the total number of missing persons. Each target $MP_{i}$ has an unknown initial position. At any given time t, its position on the 2D horizontal map is represented by the vector $p_{MP_{i}}(t)$:

(2) $$p_{MP_{i}}(t)={\left[x_{MP_{i}}(t),y_{MP_{i}}(t)\right]}^{T}$$

Its three dimensional spatial coordinate is given by $\left(x_{MP_{i}}(t),y_{MP_{i}}(t),H(x_{MP_{i}}(t),y_{MP_{i}}(t))\right)$ key assumptions for this study are that the missing person’s initial position is unknown, their movement speed is confined to a physiologically reasonable range, and it never leaves the predefined search area: $p_{MP_{i}}(t)\in S_{xy}$.

### 2.3. The Search UAV

The primary role of the UAV in this study is to detect the location of the missing person; it does not participate in any subsequent rescue or extraction procedures. The UAV is modeled with several key characteristics: it maintains a constant cruising speed, operates continuously for the duration of the designated search without needing to refuel, and can precisely follow any planned trajectory. The 3D position of the UAV at a given time t is represented by the vector $X_{U}(t)$:

(3) $$a\;=\;1,\;X_{U}(t)={\left[x_{U}(t),y_{U}(t),z_{U}(t)\right]}^{T}$$

Here, $x_{U}(t)$ and $y_{U}(t)$ denote the horizontal coordinates of the UAV on the map plane, and $z_{U}(t)$ represents its altitude.

(4) $$a=1,\;Z_{U}(t)=H(x_{U}(t),y_{U}(t))+h_{flight}$$

Here, H(x,y) denotes the terrain elevation from the DEM at horizontal location (x,y), and $h_{flight}$ is the prescribed flight height above ground level. Together they determine the UAV’s altitude at time t. The search mission is conducted during $t\in [0,T_{\max}]$, where $T_{\max}$ is the maximum mission duration.

### 2.4. Binary Detection Model

**Figure 1:**
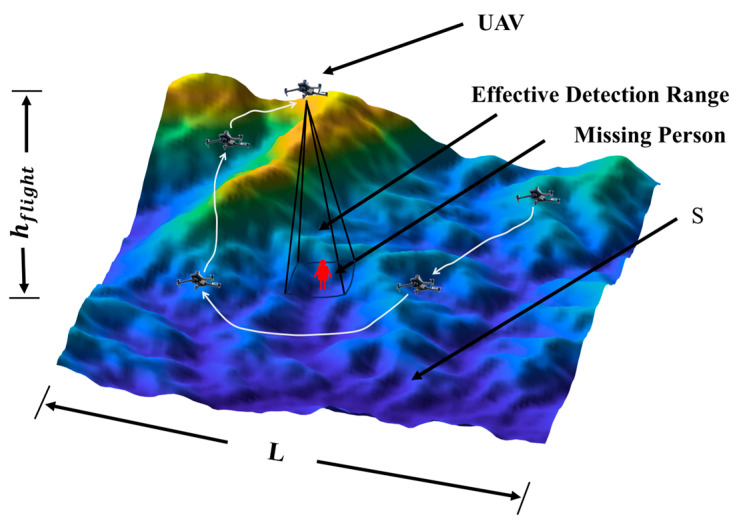
UAV missing person detection illustration.

The core objective of this study is to validate and compare different path planning strategies, not to model complex sensor physics. Therefore, we employ the widely used Binary Rectangle Model, as illustrated in Figure 1.

As illustrated in Figure 1, the UAV flies at a constant altitude above the ground and projects a square sensor footprint onto the terrain. The center of this footprint corresponds to the UAV’s horizontal position $\left(x_{U}(t),y_{U}(t)\right)$, and the side length M defines the effective detection width. A missing person located at $p_{MP_{i}}(t)$ is detected if and only if this point lies inside the shaded footprint area. This geometric relationship is formalized by Equations (5) and (6), which define the set R(t) of ground points covered by the sensor and the binary detection function D(t), respectively.

(5) $$R(t)=\{(x,y)|x_{U}(t)-\frac{M}{2}\leq x\leq x_{U}(t)+\frac{M}{2},y_{U}(t)-\frac{M}{2}\leq y\leq y_{U}(t)+\frac{M}{2}\}$$

Under this model, a detection event is a binary outcome. We formalize this with a detection function, D(t), which takes the real time positions of the UAV and the missing person as input:

(6) $$D(t)=\left\{\begin{array}{cc} 1,\; & if\;p_{MP_{i}}(t)\in R(t)\\ 0,\; & if\;p_{MP_{i}}(t)\notin R(t) \end{array}\right.$$

Here, $p_{MP_{i}}(t)={\left[x_{MP_{i}}(t),y_{MP_{i}}(t)\right]}^{T}$ denotes the horizontal position of the i-th missing person.

The function D(t) outputs 1 when the person lies inside the footprint R(t), and 0 otherwise.

In search operations, UAVs are typically equipped with thermal imagers or visible light cameras to detect missing persons. According to Johnson’s criteria, detection is classified into three levels: detection, recognition, and identification. For SAR missions, merely detecting or recognizing a heat source is insufficient. For instance, with a DJI H20T camera, flying at an altitude below 100 m allows for clear observation of human behavioral postures (e.g., waving or crouching), which is vital for assessing a missing person’s condition. It is therefore necessary for SAR operations to achieve the “identification” level of detection.

Accordingly, we set the UAV’s operational altitude at 100 m, resulting in a detection footprint of 900 m^2^. This dimension is selected to reflect the effective range required to reliably achieve the “identification” standard at a typical operational altitude. The Binary Rectangle Model is widely used in the path planning domain for this purpose. Furthermore, employing a deterministic sensor model significantly reduces the computational complexity of the simulation, enabling large scale, repeatable experiments capable of yielding statistically significant and reliable results.

## 3. Terrain-Informed Probability Modeling

### 3.1. Slope Map Generation

To effectively construct a motion model for a missing person, integrating geographic features into the map system is crucial. This is accomplished using a Digital Elevation Model (DEM), a numerical representation of surface topography that stores elevation data to accurately model terrain undulations.

**Figure 2:**
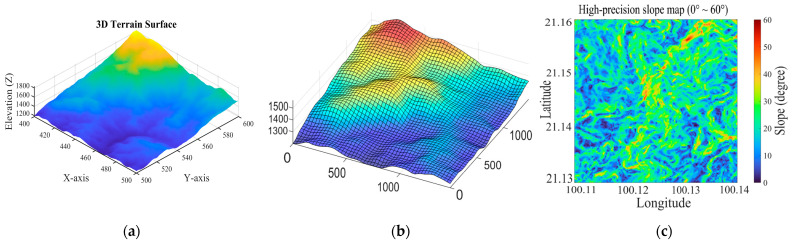
Process of establishing the mountainous environment; (**a**) original map; (**b**) mountainous grid map; (**c**) mountain slope map.

In this study, the DEM data, provided as a regular raster grid, are processed into a discrete elevation map over the study area. The continuous geographic coordinates $\left(x,y\right)$ are thus approximated by a grid, where each cell is indexed by integer coordinates $\left(i,j\right)$. To balance computational efficiency with the preservation of essential topographical features, the map is discretized into a 50 × 50 grid, where each cell corresponds to an area of approximately 30 m. This resolution is consistent with the native sampling interval of the Advanced Spaceborne Thermal Emission and Reflection Radiometer Global DEM, allowing each grid cell to represent the terrain information of a single DEM pixel. Such a configuration preserves meaningful terrain variations while avoiding unnecessary interpolation or smoothing of the elevation data, and it also maintains reasonable computational efficiency for large scale simulations. Based on this discrete representation, the east west and north south slope components, denoted by $G_{Mx}\left(i,j\right)$ and $G_{My}\left(i,j\right)$, are subsequently calculated for each grid cell using the central-difference method. Figure 2 illustrates the process of constructing a mountainous area map.

For east west slope $G_{Mx}\left(i,j\right)$, the formula is:

(7) $$G_{Mx}\left(i,j\right)=\frac{z\left(i,j+1\right)-z(i,j-1)}{2\Delta d}$$

For north south slope $G_{My}\left(i,j\right)$, the formula is:

(8) $$G_{My}\left(i,j\right)=\frac{z\left(i+1,j\right)-z(i-1,j)}{2\Delta d}$$

In these formulas, $z\left(i,j\right)$ represents the elevation value at the grid cell with vertical coordinate i and horizontal coordinate j, while Δd represents the physical spacing of the grid. These two formulas approximate the slope in the east west and north south directions, respectively, by calculating the ratio of the elevation difference between adjacent grid cells to their corresponding distance.

From these two slope components, we can derive the average slope angle $\theta \left(i,j\right)$ (in degrees). This is achieved by first calculating the gradient magnitude, which represents the total rate of slope change on the horizontal plane. The formula for the gradient magnitude is:

(9) $$\theta \left(i,j\right)=\arctan\left(\sqrt{G_{Mx}{\left(i,j\right)}^{2}+G_{My}{\left(i,j\right)}^{2}}\right)\times \frac{180}{\pi }$$

**Figure 3:**
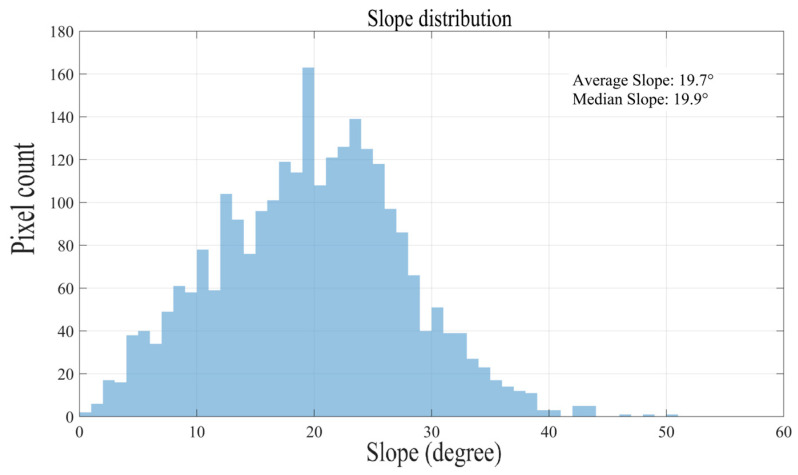
Number of grids with different slopes in the area.

Figure 2a,b show the terrain grid map before and after resampling, respectively, while Figure 2c displays the resulting slope map. In the slope map, the color gradient from blue to red corresponds to slope values ranging from 0 to 60 degrees. Figure 3 presents a histogram illustrating the distribution of these slope values across all grid cells.

The data shows that the terrain has an average slope of 19.7° and a median slope of 19.9°, indicating that moderately steep slopes are the most common feature in this area.

Based on the calculated slope angles, we classify the terrain into four categories: flat, gentle slope, steep slope, and extremely steep. Table 1 details the slope ranges for each category. This classification is foundational for the subsequent development and analysis of the motion models presented in this paper.

### 3.2. Formulation of Dynamic Missing Person Models

In search scenarios, the behavior of missing person is highly uncertain, influenced by a complex interplay of terrain, physiological condition, and psychological factors. Traditional strategies often rely on static probability maps, such as distance ring models derived from historical data, to predict a missing person’s potential location. A key limitation of these methods is the underlying assumption that the missing person is stationary from the outset of the search. However, the rapid deployment of Unmanned Aerial Vehicles means that a search can commence while the missing person is likely still mobile. Consequently, the static missing person assumption is no longer tenable. Indeed, research based on Wilderness Search and Rescue data confirms that constructing dynamic models to reflect the missing person’s continuous movement is crucial [30].

Studies on missing person behavior show that individuals attempting to rescue themselves often rely on heuristic strategies or instinctive rules of thumb, hoping to find their way to safety. Building on this, Koester analyzed numerous SAR cases and categorized the behavioral strategies of missing persons into three main types [31], which are presented in Table 2.

Building on this classification, we propose three distinct dynamic motion models that incorporate terrain influence as a core element to more accurately simulate a missing person’s spatiotemporal evolution in mountainous environments. These models are: the Terrain Constrained model, where paths are adjusted based on terrain; the Path Following model, which prioritizes movement along existing routes; and the Random Walk model, characterized by no clear directional rules [32].

(1) Terrain Constrained Model

This model simulates individuals with limited wilderness experience and orientation skills. Such individuals typically have limited physical endurance, and their movement decisions are significantly constrained by terrain slope. To conserve energy, they instinctively avoid steep areas, preferring to move along gentle slopes or flat terrain. The motion in this model is defined as a deterministic process: at each step, the missing person always chooses the path of least resistance, i.e., the one with the gentlest slope from its current position. This behavior can be quantified as follows:

(10) $$d^{*}=\arg\min\left|\tan(\theta_{\left(i,j\right)})\right|$$

(11) $$V_{t}=v_{base}\cos(\theta_{d^{*}})\;u_{d^{*}}$$

Here, $\theta \left(i,j\right)$ is the terrain slope angle computed from the DEM along that direction. The index $d^{*}$ denotes the direction with the minimum slope, i.e., the path of least resistance. The vector $u_{d^{*}}$ is the unit vector pointing along direction $d^{*}$. The parameter $v_{base}$ is the nominal walking speed on flat terrain, and the factor $\cos(\theta_{d^{*}})$ reduces the effective speed as the slope increases, resulting in the velocity vector $v_{t}$ aligned with the gentlest descent (or ascent) direction.

The chosen values are consistent with empirical studies of human walking speeds, which report typical adult walking velocities of approximately 1.1–1.7 m/s on level ground and sustained speeds below about 2 m/s on sloped terrain. Therefore, to remain within physiological limits for a lost person in mountainous terrain, the maximum speed in this and the following models is capped at 2 m/s.

(2) Path Following Model

This model is designed to characterize individuals with some wilderness experience and strong physical fitness, such as seasoned hikers. Even when lost, such individuals may retain the ability to maintain a specific direction and are capable of traversing terrain with moderate slopes (e.g., 10–25°). They exhibit a tendency to move along contour lines to avoid drastic changes in elevation, thereby minimizing energy expenditure. This motion is defined by the following equation:

(12) $$v_{t}=\mu_{t}\;v_{base}\frac{\left[-G_{My}\left(i,j\right),G_{Mx}\left(i,j\right)\right]}{\sqrt{G_{Mx}{\left(i,j\right)}^{2}+G_{My}{\left(i,j\right)}^{2}}},\;\mu_{t}\sim u(0.8,1.2)$$

In this model, the missing person tends to move approximately along contour lines. The $G_{Mx}\left(i,j\right)$ and $G_{My}\left(i,j\right)$ denote the east west and north south slope components at that cell. The vector $\left[-G_{My}\left(i,j\right),G_{Mx}\left(i,j\right)\right]$ is orthogonal to the gradient vector $\left[G_{Mx}\left(i,j\right),G_{My}\left(i,j\right)\right]$ and thus approximately tangent to the contour line. After normalization, this direction is scaled by the base walking speed $v_{base}$ and by a random factor $\mu_{t}$ drawn from the uniform distribution u(0.8,1.2), which introduces small variability in speed. The resulting velocity $v_{t}$ therefore describes motion that follows regions of nearly constant elevation. The base speed $v_{base}$ is set to 1 m/s to represent a robust travel pace.

(3) Random Walk Model

This model represents a missing person who has become completely disoriented, leading to highly unpredictable movement. Although the direction of travel exhibits no clear intentional pattern, the walking speed remains physically constrained by terrain and human mobility limits.

The direction of motion is modeled as a Markov process in which the heading at the next time step depends only on the current orientation. The initial heading angle $\phi_{0}$ is drawn uniformly from the interval (0,2π), reflecting complete uncertainty about the initial orientation. At each subsequent step, the heading evolves according to:

(13) $$\phi_{0}\sim u(0,2\pi )$$

(14) $$\Delta \phi_{t}\sim N\left(0,0.3^{2}\right)$$

(15) $$\phi_{t}=\phi_{t-1}+\Delta \phi_{t}$$

where $\Delta \phi_{t}$ models small, zero-mean reorientation events. Although the SAR literature does not report standard deviations for heading change explicitly, typical agent-based simulations employ small angular perturbations to ensure that trajectories fall between purely ballistic and purely diffusive regimes. Accordingly, we choose $\sigma_{\phi }=0.3$ rad as the default value, and we have verified through sensitivity tests that varying $\sigma_{\phi }$ within a reasonable range (0.2–0.5 rad) yields similar path tortuosity and displacement statistics. Thus, the model behavior and the comparative performance of the search strategies remain stable with respect to this parameter.

The walking speed at each step is determined by a base speed $v_{base}$ and a random scaling factor:

(16) $$v_{t}=u_{t}v_{base}\;,\;u_{t}\sim N\left(1,\;0.2^{2}\right)$$

where $u_{t}\sim N\left(1,\;0.2^{2}\right)$ is a Gaussian scaling factor that captures natural variability in walking pace due to fatigue, terrain difficulty, or psychological state. The instantaneous speed is given by $v_{t}=u_{t}v_{base}$ and is capped at 2 m/s to maintain physical realism. The resulting velocity vector is then:

(17) $$v_{t}=v_{t}\left[\begin{array}{c} \cos\phi_{t}\\ \sin\phi_{t} \end{array}\right]$$

which specifies the direction and magnitude of movement at time t.

**Figure 4:**
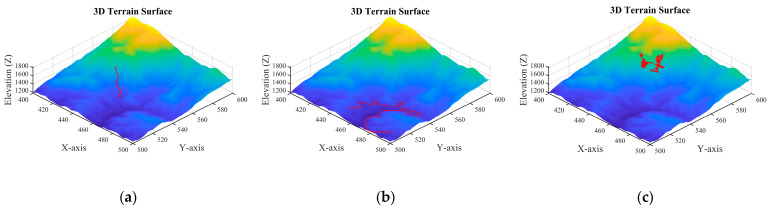
Schematic diagrams of the motion trajectories of three typical models: (**a**) terrain constrained type, (**b**) path following type, and (**c**) random walk type.

While the three models detailed above can simulate a missing person’s motion, as illustrated by their respective trajectories in Figure 4, a critical challenge remains: the missing person’s initial position is unknown. Therefore, to effectively launch the UAV search operation, we must first estimate the missing person’s probable locations by leveraging prior terrain information.

### 3.3. Generating the Probability Map

As established in Section 3.1, we first generate a slope map for the search area. Using the slope data derived for each grid cell, we then construct a global a priori probability map to represent the initial likelihood of the missing person’s presence across the region.

This map is founded on the key behavioral assumption that lost individuals tend to avoid steep terrain and gravitate towards gentler slopes. To formalize this relationship, we develop a model that couples slope with probability. For any given grid cell (i,j), its initial probability of presence, $P_{0}(i,j)$, is defined as follows:

(18) $$P_{0}(i,j)=\frac{e^{-\alpha \theta_{ij}}}{\sum e^{-\alpha \theta_{ij}}}$$

(19) $$\sum_{i,j}P_{0}(i,j)=1$$

In this formulation, the term $\theta_{ij}$ represents the average slope of each grid (i,j), and the coefficient α controls the extent to which steep terrain reduces the prior probability of target presence. Although α is implemented as a fixed constant, its selection is guided by well-established observations regarding human movement in mountainous environments. Missing persons usually remain in gentle terrain where movement requires less effort and where the risk of falling is low. As the slope becomes larger, the difficulty and danger of moving across the terrain increase quickly, and the likelihood of a person entering such areas decreases accordingly.

To reflect this behavior, an exponential decay function is employed because it produces a probability that decreases rapidly as the slope increases. This produces a distribution in which gentle terrain retains relatively high probability, moderately sloped regions receive reduced probability, and very steep areas are strongly suppressed. The value α of is chosen through preliminary testing of the overall performance of the search framework under different settings. The selected value provides an appropriate balance between penalizing steep terrain and maintaining enough probability in moderately sloped regions so that the search process remains stable and effective.

**Figure 5:**
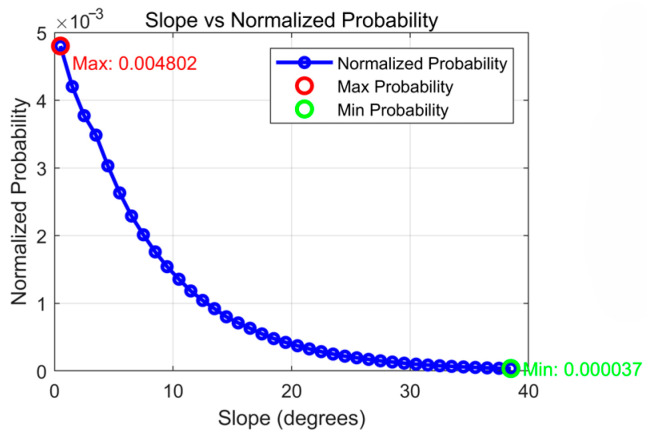
Probability corresponding to different slopes.

This relationship is visualized in Figure 5, which confirms that the probability is highest on flat terrain (a slope of 0°) and decays exponentially as the terrain becomes steeper. The curve shows a sharp decline in probability as the slope increases from 0° to 15°, and by 30°, the probability value is negligible, closely mirroring the tendency of a missing person to avoid hazardous areas.

**Figure 6:**
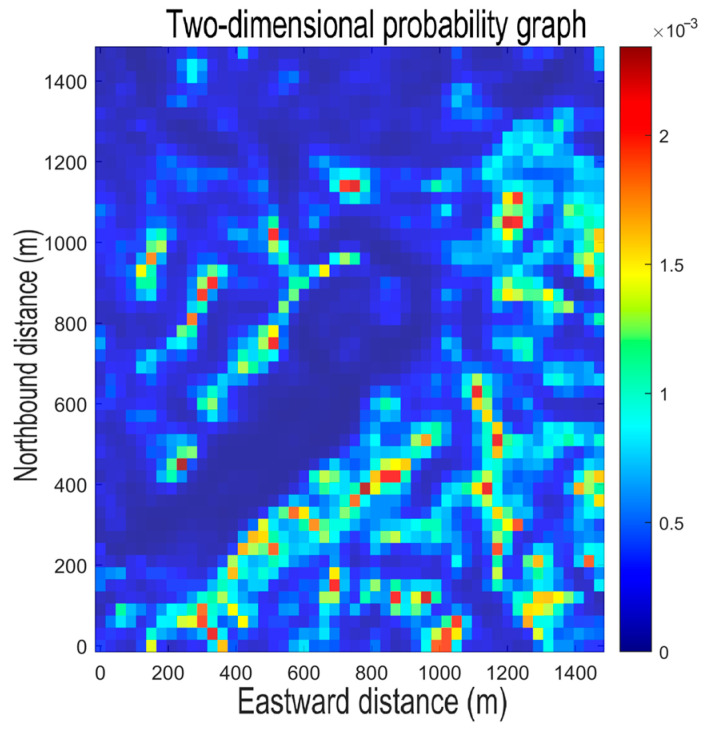
Initial probability map of the region.

Figure 6 shows the initial probability map of the region generated through this process. As indicated by the color bar, warmer colors such as red and yellow correspond to areas with a high probability of the missing person’s presence (representing gentle slopes), while cooler, dark blue colors represent areas of low probability (representing steep slopes).

The slope-based a priori probability map is established before the search begins. Once the UAV commences its mission, this map becomes dynamic, and the probability of each grid cell is updated in real time to reflect new information. Our update rule relies on the binary detection model, which assumes perfect detection within the UAV’s sensor footprint. Therefore, when a grid cell is searched at time $t_{0}$ with a negative result (no missing person found), its probability of containing the missing person decays instantaneously. However, because the missing person is mobile and may reenter a cleared area, this probability must also recover over time. This dynamic process of probability decay and recovery is described by the following:

(20) $$P(t)=\varepsilon +(P(0)-\varepsilon )[1-e^{-\lambda \left(t-t_{0}\right)}]$$

This equation governs the dynamic update of a cell’s probability, P(t), through a process of decay and recovery based on its original a priori probability, P(0). When a search of the cell is completed at time $t_{0}$ with a negative result, its probability immediately decays to ε, a very small positive value (in the ideal case of a perfect sensor, ε would be 0). Subsequently, the probability recovers exponentially toward its initial value. The rate of this recovery is controlled by λ, the probability recovery constant, where a larger value signifies a faster recovery. This feature is used to model a missing person with higher mobility, reflecting the increasing chance that it could have reentered the previously searched area.

## 4. Unmanned Aerial Vehicle Search Path Planning Algorithm

In this section, we introduce our novel path planning algorithm, which is based on the dynamic slope probability map and an iterative search framework.

### 4.1. Iterative Search Trajectory Planning

Building on the dynamic probability mechanism and missing person models developed in Section 2, this section details our iterative and adaptive UAV search algorithm. Traditional path planning algorithms, such as A*, are designed to find an optimal path between a known start and endpoint. In a SAR task, however, the missing person’s location is the central unknown. This means a search trajectory cannot be generated by a single, static plan but requires a dynamic approach.

To this end, we propose an iterative search framework that uses incremental waypoints. This framework decomposes the complex global search mission into a sequence of short term, targeted path planning sub tasks. The core idea is as follows: in each iteration, the algorithm first selects a high potential subgoal based on the current global probability map. It then invokes a modified A* algorithm to plan an optimal path from the UAV’s current position to this subgoal. The UAV executes its search along this path, updating the probability map in real time. Upon completing the path or meeting predefined replanning criteria, the system reassesses the situation, selects a new subgoal, and initiates the next planning cycle. This process repeats until the missing person is located or the search time expires. Our proposed algorithm thus functions as a closed loop “perceive plan act” system.

The A* algorithm serves as the foundation of this framework, guiding the expansion of search nodes via an evaluation function, $f_{n}$.

(21) $$f_{n}=g_{n}+h_{n}$$

**Figure 7:**
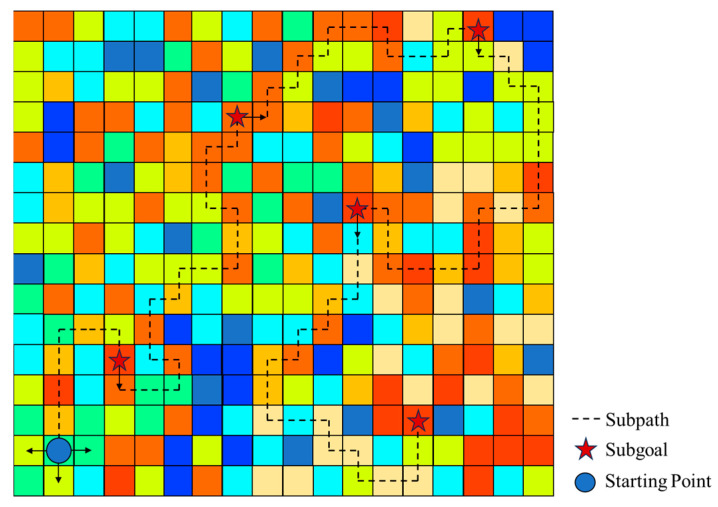
Principle diagram of A* search with subgoal strategy.

The A* evaluation function, $f_{n}$, consists of two components: $g_{n}$, which is the actual cost of the path from the start node to the current node n, and $h_{n}$, which is the estimated cost from node n to the goal, as determined by a heuristic function. At each step, the algorithm expands the node from the priority queue that has the lowest $f_{n}$ value, a process that guarantees the path’s optimality. In the grid environment of this study, each grid cell is treated as a node. As shown in Figure 7:

As shown in Figure 7, the color of each node corresponds to its probability value. Based on the color convention from Figure 6, warmer colors like red and orange represent high probability areas, whereas cooler colors like blue denote regions of low probability. This probability value, in turn, influences the node’s associated cost. Arrows indicate the direction of selectable paths. The algorithm proceeds by selecting the node with the lowest value from the cost evaluation function to explore next, thereby ensuring the planning is always guided in the most promising direction.

### 4.2. Subgoal Selection Strategy

In our iterative framework, each planning cycle begins with selecting a subgoal from the global map, a critical step that determines the search direction for the next phase. To guide this process, we construct a comprehensive scoring matrix where the value for each grid cell is determined by the formula $M_{score}(i,j)$. This score evaluates the cell’s potential to serve as the next subgoal. The most influential factor, the cell’s current probability value P(i,j), biases the search toward regions where the missing person is most likely to be present.

(22) $$M_{score}(i,j)=P(i,j)\times R_{\exp lore}(i,j)\times D_{sparse}(i,j)$$

In addition to the probability factor, the scoring matrix includes two other terms to encourage comprehensive searching. The first is an exploration reward, $R_{\exp lore}(i,j)$, which incentivizes the algorithm to explore unvisited or infrequently visited regions, thereby preventing premature convergence to a local optimum. The value of this reward is inversely proportional to the node’s historical visit count. The second factor is a spatial sparsity ratio, $D_{sparse}(i,j)$, which suppresses redundant searches in areas with high visit density. Its calculation is defined by the following formula:

(23) $$D_{sparse}=\frac{1}{1+{\left[G_{\sigma }*V\right]}^{0.8}}$$

In this formula, V is a matrix that records the history of visited locations, and $G_{\sigma }$ is a Gaussian smoothing kernel. A visit density score is obtained by performing a convolution operation between these two terms. This process results in a map where regions with a higher visit density receive a lower spatial sparsity score, $G_{\sigma }*V$.

Once the comprehensive scoring matrix is calculated, the subgoal selection proceeds in two stages. First, we form a candidate set C by selecting all grid cells whose scores exceed a given threshold τ (top 10% of scores). Second, the subgoal for the current iteration is determined using a weighted Monte Carlo sampling method applied to this set C, where each candidate’s selection probability is proportional to its score.

(24) $$C=\{(i,j)M_{score}(i,j)\geq \tau \},\tau =Percentile(M^{+},90\%)$$

(25) $$p_{select}(i,j)=\frac{M_{score}(i,j)}{\sum_{(i,j)\in c}M_{score}(i,j)}$$

Here, $M_{score}(i,j)$ denotes the comprehensive score assigned to grid cell (i,j), and $M^{+}$ represents the set of all score values over the map. is the probability of selecting cell $\left(i,j\right)$ as the subgoal, and the denominator $\sum_{(i,j)\in c}M_{score}(i,j)$ normalizes these probabilities so that they sum to one over all candidates.

This stochastic strategy is adopted because, in many practical search scenarios, the scoring matrix contains large areas with almost identical values, particularly during the early stage of the mission. A deterministic rule that always selects the highest score would make the planner behave greedily, revisiting the same limited set of cells and becoming highly sensitive to noise or small fluctuations in the probability map. In contrast, weighted Monte Carlo sampling introduces a form of controlled randomness. Cells with higher scores are still more likely to be selected, while those with lower scores retain a small probability of being chosen. This mechanism promotes a more balanced interaction between exploitation and exploration, helping the UAV avoid becoming trapped in local regions and improving the overall robustness of the search process.

### 4.3. Design of the Heuristic Function and Cost Function

The traditional A* algorithm is designed to find the shortest path in a static environment, where traversal cost is typically a function of distance. Our task, however, is fundamentally different: to efficiently find a mobile missing person with an unknown location in a dynamic information landscape.

Within our iterative framework, the objective of each path planning sub task is therefore not to find the geometrically shortest route, but rather to generate a path that maximizes the probability of locating the missing person. This redefinition of an “optimal” path requires that our heuristic and cost functions move beyond purely distance calculations. They must instead integrate multiple dimensions of information, including terrain data, search history, and the missing person’s probability of presence.

For each path planning sub task, the cost function for any given node n is defined as follows:

(26) $$g(n,n^{\prime })=\beta_{1}{\left\|n^{\prime }-n\right\|}_{2}+\beta_{2}e^{\alpha p\left(n^{\prime }\right)}+\beta_{3}\sqrt{v(n^{\prime })}+\beta_{4}\sum_{k\in K}c_{k}(n^{\prime })$$

The actual cost $g(n,n^{\prime })$ to reach a node n’ from the node n is defined as a comprehensive weighted sum that quantifies the overall cost of a path. While retaining the standard Euclidean distance to measure physical travel cost, we introduce three additional critical cost factors: a slope penalty, a visit penalty, and a coverage density penalty.

The slope penalty uses an exponential function rather than a linear one to more strongly penalize movement into high slope areas. This reflects the reality that as slope increases from 20° to 40°, for example, the traversal difficulty and risk grow nonlinearly. This design has a dual benefit: it guides the UAV away from terrain that could endanger its safety, and more importantly, it creates a strategic synergy with the “slope avoidance” behavior of the missing person, coupling the UAV’s search focus with the missing person’s most likely locations.

The visit penalty is designed to increase the algorithm’s exploratory behavior. It prevents the search from becoming trapped in small loops or repeatedly traversing areas that have already been sufficiently explored simply because of low local costs. Each time the UAV revisits a node, its traversal cost increases, thus incentivizing the algorithm to seek out new, unvisited paths with higher informational value.

The coverage density penalty complements and refines the visit penalty. While the visit penalty $v(n^{\prime })$ applies to individual nodes, the coverage density penalty is designed to prevent the search path from becoming too spatially clustered. It employs a Gaussian coverage density kernel $c_{k}(n^{\prime })$ centered on historical path points, which creates a “repulsion field” around the flown trajectory. If a candidate node for a new path falls within this field, its cost is increased. This effectively encourages new search paths to maintain spatial separation from previous ones, promoting a more uniform and efficient area coverage.

In Equation (26), the coefficients $\beta_{1}\sim \beta_{4}$ represent the relative importance of path length, search behavior, historical visitation, and coverage density, and they satisfy the normalization constraint $\beta_{1}+\beta_{2}+\beta_{3}+\beta_{4}=1$. Their values are determined in two steps. First, we initialize the weights to ensure that each component contributes meaningfully to the overall cost. Then, we refine these initial values through a localized parameter search, which adjusts the coefficients to achieve a balanced and effective performance in the search task.

The heuristic function, h(n), is designed to estimate the future cost from the current node n to the subgoal $n_{g}$. Its design is critical as it directly governs the search’s efficiency and direction.

(27) $$h(n)={\left\|n-n_{g}\right\|}_{1}\times (1-\gamma_{1}P(n)+\gamma_{2}\frac{1}{1+\log(1+v(n))})$$

The first component of our heuristic, $1-\gamma_{1}P(n)$, leverages information from the dynamic probability map. When the probability of the missing person being present at node n, denoted as P(n), is high, this term becomes very small. The resulting decrease in the estimated cost to go creates a strong attraction that biases the search trajectory toward the most promising regions, thereby making the search more purposeful. The weighting coefficient $\gamma_{1}$ regulates the strength of this probabilistic attraction and determines how aggressively the planner exploits areas with high posterior probability.

The second component, v(n) serves as the primary exploration driver. Its formulation incentivizes the search to explore regions with little or no prior visitation. For unvisited nodes (i.e., visit count v(n) is 0, the reward term 1+log(1+v(n)) evaluates to a value close to $\gamma_{2}$, substantially reducing the heuristic estimate and producing a strong incentive for exploration. As the visit count increases, the term grows logarithmically. The logarithmic scaling ensures smooth growth and prevents disproportionately large penalties for heavily revisited nodes. As a result, h(n) gradually approaches 1, causing the exploration incentive to diminish over time and preventing repetitive revisiting of already explored areas.

The two weighting coefficients, $\gamma_{1}$ and $\gamma_{2}$, determine the balance between these complementary mechanisms. After normalizing each heuristic component to a comparable numerical range, $\gamma_{1}$ controls the level of exploitation of high probability regions, while $\gamma_{2}$ governs the degree of exploration of unvisited areas. By appropriately tuning these coefficients, the planner achieves a dynamic and adaptive balance between exploiting reliable information and exploring unknown possibilities. This balance is essential in dynamic search and rescue missions, where the missing person’s location may change over time. For parameter values, see the Appendix A Table A1.

### 4.4. Algorithm Flow

**Figure 8:**
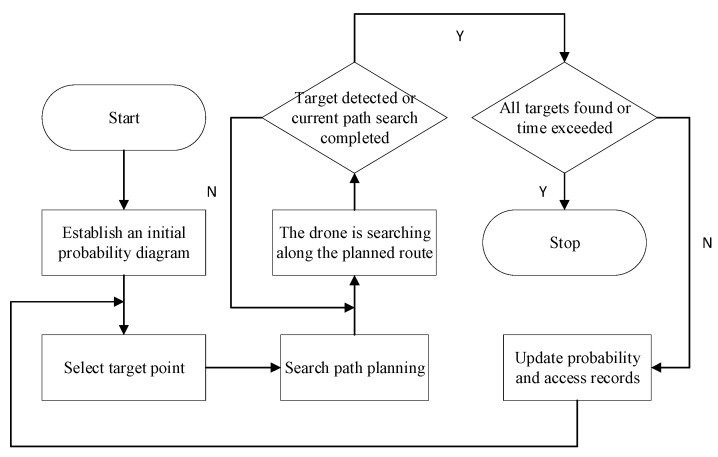
Overall algorithm flow.

The overall strategy of the proposed algorithm is shown in Figure 8:

Initially, a slope probability map is generated from the environmental data, and the UAV is deployed at a safe starting point. The UAV then enters an iterative search cycle where, at the beginning of each iteration, a high scoring node is selected as a subgoal. A planning algorithm, guided by a heuristic function that fuses the attraction of high probability areas with a reward for exploring unvisited regions, generates a trajectory to that subgoal. Concurrently, as the UAV moves along the planned path, the dynamic probability map and the record of visited nodes are updated in real time. This cycle of subgoal selection, planning, and execution repeats until the process terminates, which occurs when either all missing persons are successfully located or the maximum allotted search time is exceeded.

## 5. Simulation Experiments

In this section, we quantitatively evaluate the performance of our proposed strategy through a series of simulation experiments. To ensure realism and validity, all simulations are based on real-world geographical data and executed on a standardized computing platform.

### 5.1. Experimental Setup

**Figure 9:**
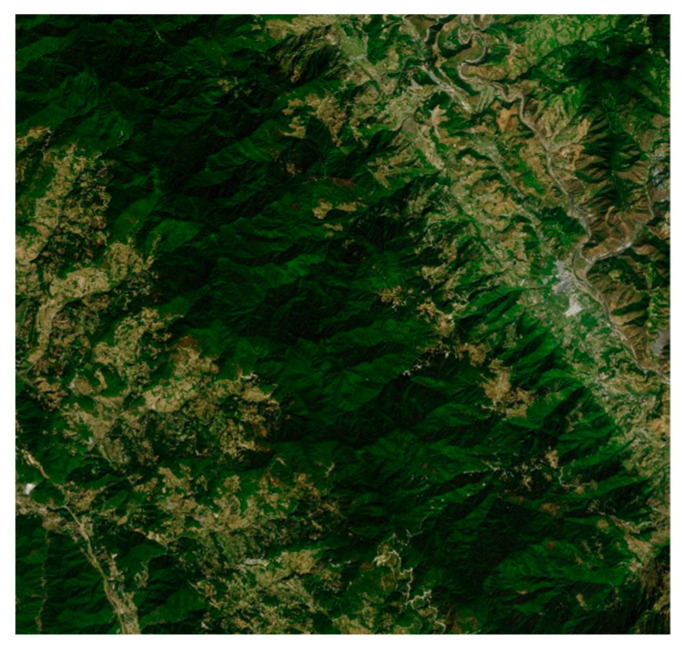
Experimental satellite map.

(1) Geographic environment:

As shown in Figure 9, the simulation environment is a mountainous area in Yunnan Province, China, with geographical coordinates ranging from 24.0° N to 24.1° N and 101.3° E to 101.6° E. We use Digital Elevation Model (DEM) data from ASTER GDEM. For the purpose of our simulation experiments, we selected a specific 1.5 km × 1.5 km sub-region, covering an area of approximately 2.25 km^2^. This search area was then discretized into a 50 × 50 grid map, where each cell represents a 30 m × 30 m area. The elevation within this region varies from 0 to 1600 m.

(2) Simulation parameters:

All experiments were run on a PC equipped with an AMD R5 5600 CPU and 32 GB of RAM, using MATLAB R2023b. The maximum duration for each search mission is set to 2500 steps. This value is based on the time required for a traditional traversal algorithm (e.g., a lawnmower search) to achieve full coverage of the area. To account for the randomness of the missing persons’ initial positions, each algorithm under comparison was executed for 2000 independent trials to obtain statistically significant performance metrics.

(3) UAV and missing person parameters:

A single UAV performs the search at a cruise altitude of 100 m above ground level. This operational altitude is selected based on the effective range of thermal imaging systems, and the UAV’s sensor footprint is assumed to cover one grid cell per time unit. The UAV’s cruise speed is set to be ten times the base movement speed of the missing persons.

To comprehensively evaluate the algorithm’s performance in a complex scenario, the simulation is configured with multiple coexisting missing persons. Accordingly, the environment contains three independent mobile missing persons, each corresponding to one of the behavioral models developed in Section 2: Terrain Constrained, Path Following, and Random Walk.

(4) Performance Metrics

Performance is evaluated using two primary metrics:

Search Success Ratio: The percentage of trials (out of 2000) in which all three missing persons are successfully located within the time limit of 2500 units.

Average Elapsed Time: Calculated only for the successful trials, this metric represents the average number of time units (i.e., steps) from the start of the mission until the final missing person is found. It serves as a measure of search efficiency.

### 5.2. Ablation Study

To assess the independent roles of probability guidance and the exploration reward, an ablation study was conducted using four configurations. The complete SPS method, which includes both modules, serves as the reference.

A second configuration removes probability guidance but retains the exploration reward. A third configuration removes the exploration reward while keeping probability guidance. A fourth configuration removes both modules and therefore represents a geometry driven baseline. The results of these four configurations are summarized in Table 3.

The results reveal clear differences among the four variants. The complete SPS method achieves the highest success ratio and the most reliable detection of all three targets. The removal of probability guidance reduces the algorithm’s ability to move toward high likelihood regions and leads to a marked decline in overall success. The removal of the exploration reward also degrades performance. Although it preserves the global directional cues provided by the probability map, it loses the ability to avoid redundant revisits, which reduces both coverage efficiency and detection stability. When both modules are removed, the search degenerates into a purely reactive be-haviour. This configuration exhibits the lowest success ratio and the weakest detection ability for all targets.

### 5.3. Performance Comparison

We compare our proposed algorithm against four baseline methods: a lawnmower search, a spiral search, a random search, and the traditional A* algorithm. To establish a clear point of comparison, the traditional A* algorithm baseline was implemented as an iterative greedy strategy. It operates in cycles, similar to the proposed SPS algorithm. However, its decision making at each stage is simplified: at the beginning of each planning iteration, it adopts a purely greedy approach by selecting the single grid cell with the current highest probability as its subgoal. The path to this subgoal is then computed using a standard A* implementation, where both the cost and heuristic functions are based solely on Euclidean distance.

**Figure 10:**
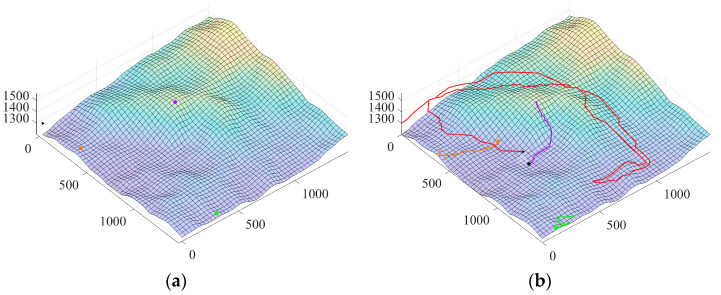

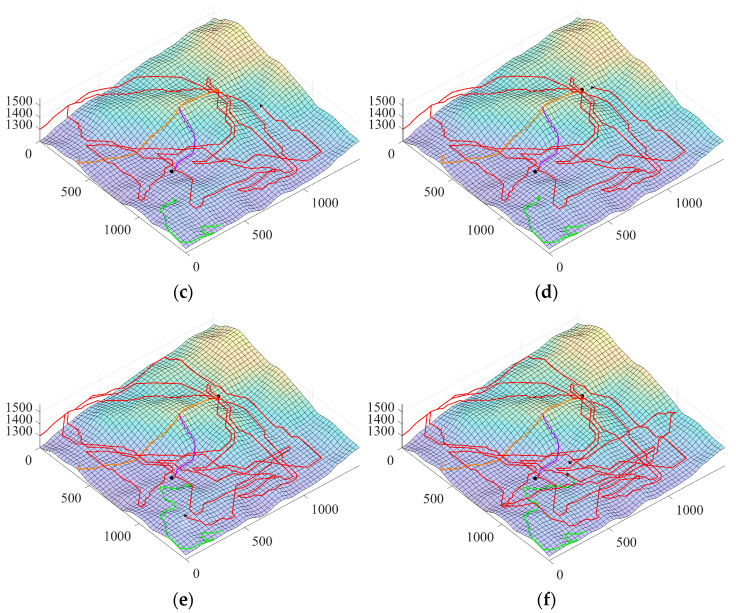
Schematic diagram of the SPS algorithm: (**a**) shows the UAV before search begins; (**b**) shows the UAV has detected the first missing person; (**c**) shows the UAV at 400 steps; (**d**) shows the UAV has detected the second missing person; (**e**) shows the UAV at 500 steps; (**f**) shows the UAV has detected the third missing person, and the search ends.

**Figure 11:**
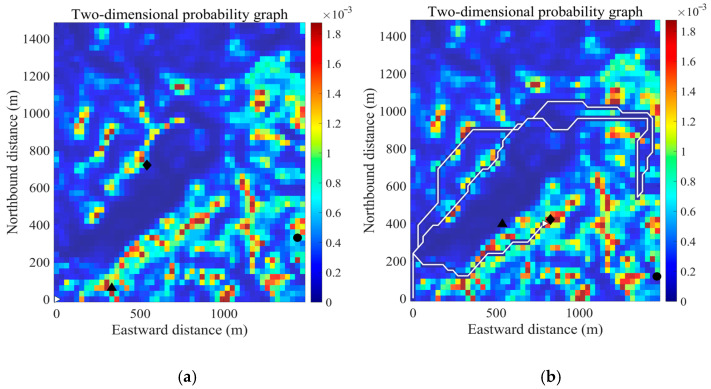

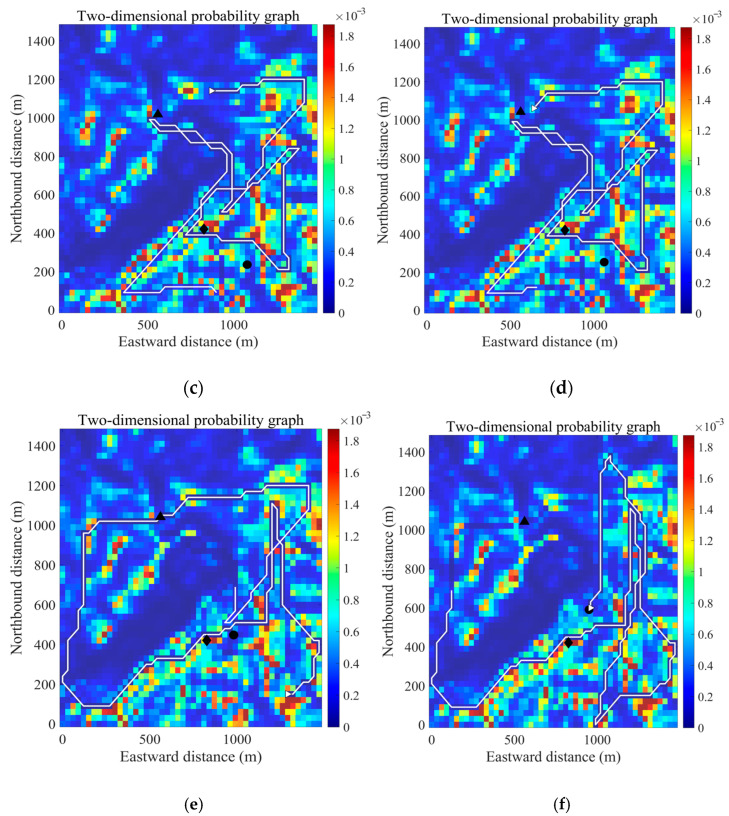
The dynamic probability map during a search mission. The white line represents the UAV’s search path, black triangles, black diamonds, and black circles represent three types of missing person locations. The probability values within the cells along this path decrease sharply after being searched and then gradually recover over time. (**a**) shows the UAV before search begins; (**b**) shows the UAV has detected the first missing person; (**c**) shows the UAV at 400 steps; (**d**) shows the UAV has detected the second missing person; (**e**) shows the UAV at 500 steps; (**f**) shows the UAV has detected the third missing person, and the search ends.

Figure 10 illustrates a simulated search mission, where the red line represents the UAV’s trajectory. The movement paths of the three missing persons are shown in purple, green, and orange. Their locations are marked by black dots when they are detected. Figure 11 shows the search process of the UAV in the 2D probability map.

**Figure 12:**
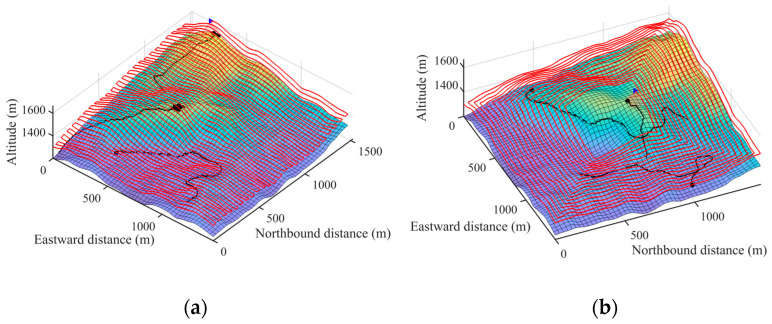
Schematic diagrams of reciprocating and spiral search patterns, (**a**) represents lawnmower search, and (**b**) represents spiral search.

For traditional search strategies, UAVs primarily employ predefined paths, such as spiral or parallel line patterns. Figure 12 illustrates two of these common approaches: the reciprocating search and the spiral search.

**Figure 13:**
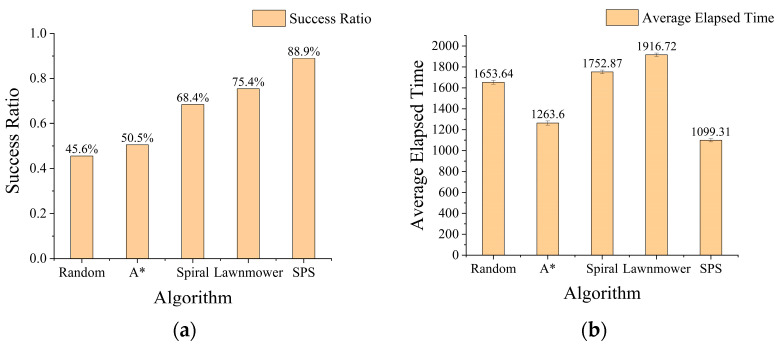
Comparison of total success rates and average search times for all strategies, (**a**) shows the success rate comparison bar chart, and (**b**) shows the average number of steps bar chart.

The overall simulation results, summarized in Figure 13, demonstrate the superior performance of the proposed SPS algorithm. The SPS method not only achieved the highest search success rate at 88.9% but also recorded the shortest average search time of 1099.31 steps. Among the traditional coverage strategies, the Lawnmower pattern (75.4%) was more effective than the Spiral pattern (68.4%), though both were significantly less efficient than the proposed method. The Random and traditional A* search strategies yielded the poorest performance, with success rates of 45.6% and 50.5%, respectively.

All performance statistics are computed over 2000 independent trials. The average search time of 1099.31 steps, calculated from 1779 successful runs, has a standard error of 15.7 and a 95% confidence interval of [1068.5, 1130.1]. These narrow intervals relative to the nominal values indicate that both the estimated success rate and the average search time are statistically stable and reliable.

### 5.4. Analysis of Missing Person Model Influence

**Figure 14:**
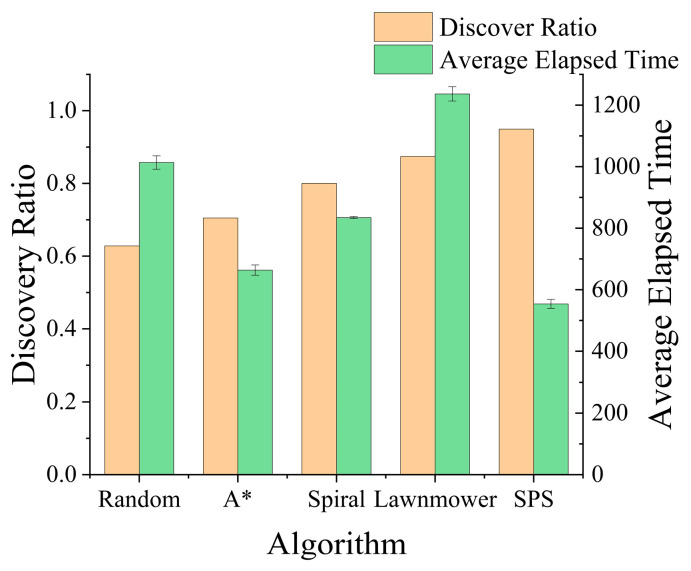
Comparison of performance for various algorithms against the Path Following Model.

As illustrated in Figure 14, the Path Following model presented the greatest challenge, resulting in the lowest discovery rates for most algorithms. The success rates for traditional strategies against this missing person type ranged between approximately 60% and 80%. This difficulty stems from this model having the highest mobility and the most extensive movement range, which makes it particularly elusive for conventional search patterns to track effectively. In contrast, our proposed strategy demonstrated a pronounced advantage in this scenario. By adaptively expanding its search based on terrain information, our algorithm covers potential travel routes far more rapidly than other methods. This superior effectiveness against the most challenging missing person type is a key factor contributing to our strategy’s significantly higher overall success rate. Furthermore, as shown by the green bar in Figure 14, the SPS algorithm achieved this high success rate with a substantially lower average search time, demonstrating its superior efficiency in tracking highly mobile missing persons.

**Figure 15:**
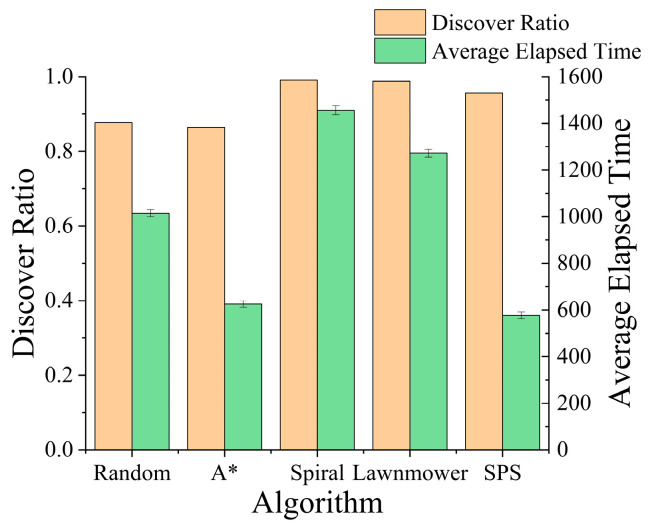
Comparison of performance for various algorithms against the Terrain Constrained Model.

The search for terrain constrained missing persons represents a scenario where all strategies achieve high success rates, each exceeding 83%. This high performance is expected, as the missing person’s movement is heavily restricted by the terrain, confining it to a relatively small activity range. However, while the success rates are comparable, Figure 15 highlights a stark contrast in search efficiency. Our proposed SPS algorithm consistently located the missing person in significantly fewer steps on average than any other method, particularly outperforming the traditional coverage patterns. This result demonstrates that even in less challenging scenarios, the intelligence driven approach of SPS provides a crucial advantage, leading to substantially faster mission completion.

**Figure 16:**
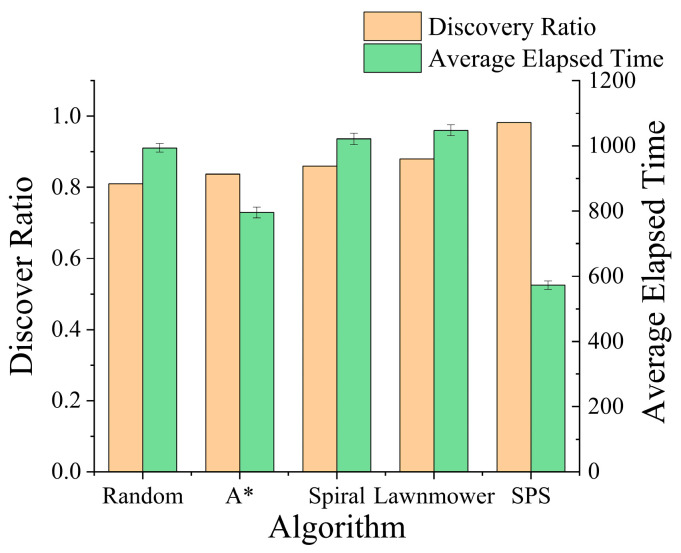
Comparison of performance for various algorithms against the Random Walk Model.

The search success rate for random walk model was slightly lower than for terrain constrained model, though both remained above 80%. While these missing persons are less restricted by terrain, their speed is still influenced by it, and their tendency to retrace previous paths similarly limits their movement range. Crucially, Figure 16 shows that the SPS algorithm’s efficiency advantage is pronounced in this scenario as well. It consistently located the missing person in the fewest average steps, once again validating the superiority of its dynamic, probability guided search strategy over fixed patterns or purely greedy methods.

### 5.5. Parameter Sensitivity Experiment

(1) Analysis of the Slope Sensitivity Coefficient α

**Figure 17:**
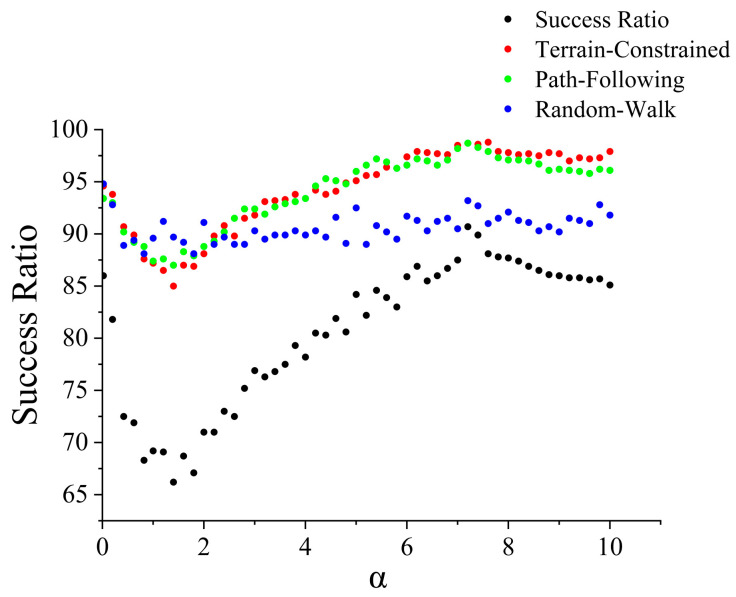
Missing person success rate line chart under different parameter α.

The key parameter of the proposed search strategy model, the slope sensitivity coefficient α, significantly influences the algorithm’s search behavior and final performance. To validate the rationality of the selected parameters and gain a deeper understanding of their impact on the model, this section conducts parameter sensitivity analysis. We systematically test the effects of different parameter values on search success rate and average search time, and investigate the model’s robustness and reliability. The parameter α controls the suppression strength of slope on the missing person existence probability in the initial probability map. If α is too small, the terrain information is not fully utilized, and the algorithm approaches random search; If α is too large, the algorithm may become overly confident in flat areas and ignore missing persons moving to other regions. The test range is set to 0–10. For each value of α, 2000 search tasks are tested. The experimental results are shown in Figure 17, where the scatter plot illustrates the overall success rate of the algorithm (black points) and the success rates of the three missing person motion models—Terrain-Constrained, Path-Following, and Random-Walk (colored points).

Based on the parameter sensitivity experiments, the slope sensitivity coefficient α has a significant and nonlinear influence on the performance of the search algorithm. When α approaches zero, the probability map becomes nearly uniform, and the search strategy degenerates into a broad traversal-like exploration that still achieves a relatively high success rate. As α increases slightly, weak but unstable probability cues begin to distort this original pattern, producing a conflicted search behavior that leads to a sharp decline in success rate, reaching its minimum around α ≈ 1.5. When α increases further, the probability map becomes more discriminative and provides a reliable guide for the search process. This targeted behavior improves efficiency and causes the success rate to rise steadily until it reaches its peak at α = 7.2. This value offers a balanced trade-off between terrain discrimination and adequate search coverage. Therefore, α = 7.2 is adopted in this study.

(2) Analysis of the Probability Recovery Constant λ

**Figure 18:**
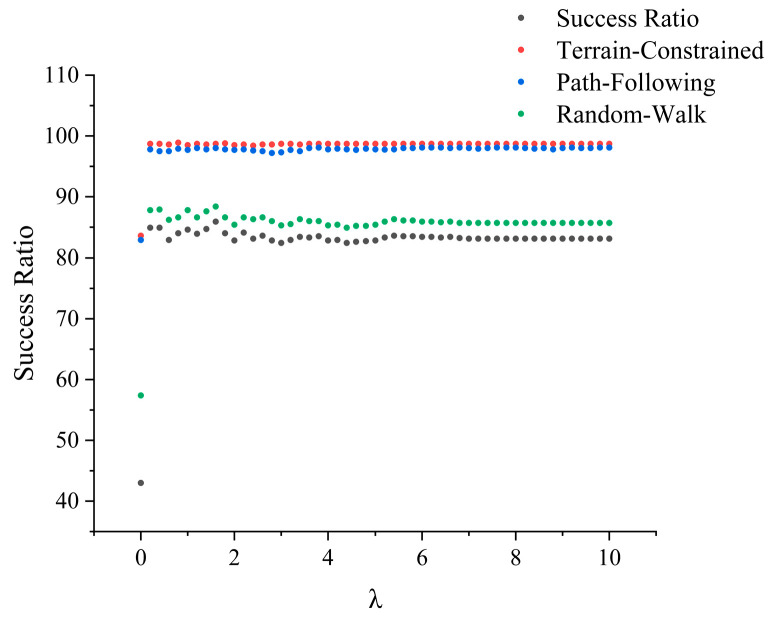
Missing person success rate line chart under different parameter λ.

The second critical parameter analyzed is the probability recovery constant λ, which is central to the dynamic probability update mechanism. This parameter governs the rate at which the probability of a previously searched area recovers, directly reflecting the algorithm’s intrinsic assumption about the missing person’s mobility. The experimental results are shown in Figure 18, where the scatter plot illustrates the overall success rate of the algorithm (black points) and the success rates of the three missing person motion models—Terrain Constrained, Path Following, and Random Walk (colored points).

The analysis reveals two key findings. First and foremost, in stark contrast to its high sensitivity to the α parameter, the SPS algorithm demonstrates exceptional robustness with respect to λ. A prominent finding is that enabling the probability recovery mechanism is critical to success. When λ is close to zero, the success rate is extremely low, as the algorithm fails to account for the missing person’s dynamic nature. However, once λ increases to a reasonable value (e.g., >1.0), the success rate for all missing person models rises dramatically and remains consistently high. This is a significant result, as it indicates that the algorithm’s effectiveness is not contingent on precise tuning of λ, highlighting its practical utility in real-world scenarios.

Second, while the overall performance is stable, subtle trends reveal a clear correlation between the optimal λ value and the missing person’s mobility characteristics. For the highly mobile Path Following and predictable Terrain Constrained models, the performance curves stabilize at an exceptionally high success rate and are largely insensitive to λ once it is in the effective range. However, for the highly uncertain Random Walk model, the success rate peaks at a specific λ value before slightly declining. This suggests that a balanced recovery rate is crucial for unpredictable missing persons: a recovery that is too slow cannot keep up with the missing person’s movements, while one that is too fast may cause inefficient repetitive searching.

## 6. Discussion

An analysis of detection rates shows the algorithm was effective against all three missing person types. Performance was highest for terrain constrained missing persons—an expected outcome, as the algorithm’s “gentle slope preference” perfectly aligns with this missing person’s “steep slope avoidance.” In contrast, the rate was lower for path following missing persons due to their larger movement range. This suggests adapting the strategy to the missing person’s profile; for an experienced hiker, increasing the exploration reward would help expand the search area more quickly. Finally, the high detection rate for random walk missing persons validates the importance of exploring low probability areas, a strategy that can be enhanced by lowering the slope sensitivity to prevent the algorithm from focusing too heavily on gentle slopes.

The traditional A* algorithm, when implemented with a greedy strategy, was one of the methods with the worst performance, achieving a success rate much lower than the simple Lawnmower pattern. This failure stems from its purely exploitative nature, which repeatedly guides the search back to the same high probability zones. Because the strategy fails to adequately explore new areas where a mobile missing person may have traveled, it becomes trapped in local optima and proves unsuitable for the dynamic nature of the search problem.

Despite the effective results of the proposed strategy, it is important to acknowledge several limitations that present avenues for future research:

(1) Behavior Model Complexity: Although the three behavioral models used in this study are representative and capture common movement tendencies, they do not fully reflect the diversity of real missing-person behaviors. In real search scenarios, an individual’s decisions are influenced by fatigue, stress, injury, terrain familiarity, and environmental cues, which are far more complex than the simplified rules used here. Future work will incorporate more detailed statistical and psychological data from actual search-and-rescue records to construct richer, more data-driven behavior models that can better describe different categories of missing persons and environmental contexts. 

(2) Idealized Sensor Model: The current sensor model is deterministic and does not explicitly account for real-world challenges such as terrain occlusion [33], vegetation cover, limited field of view, false alarms, and missed detections. These factors may significantly influence the effectiveness of aerial search. Future research will incorporate more realistic perception models that consider line-of-sight constraints, probabilistic detection functions, and sensor performance degradation under complex mountain conditions, enabling the planner to reason directly about perception uncertainty.

(3) Real-World Deployment: Future work will therefore aim to implement the algorithm on actual UAV platforms, integrate hardware in the loop testing, and conduct field trials in representative mountainous terrain. These efforts will help assess the robustness of the method under realistic operational conditions and support its transition toward practical search and rescue applications.

## 7. Conclusions

This paper addressed the challenge of inefficiently locating dynamic missing persons in mountain SAR by proposing an adaptive UAV search strategy that integrates terrain slope information with dynamic missing person modeling. Our approach quantifies topographical features into a priori prediction of a missing person’s location by constructing a novel slope probability map. This map, in turn, guides a modified iterative A* search algorithm, which uses customized cost and heuristic functions to achieve a dynamic balance between safety, exploration, and goal orientation.

Comprehensive simulation results demonstrated that our proposed strategy significantly outperforms traditional coverage-based and conventional heuristic searches in both search success rate and average search time. This study indicates that leveraging environmental a priori information and missing person behavior patterns is key to advancing beyond the limitations of traditional search methods. The findings provide both a theoretical basis and a practical algorithmic framework for the development of future intelligent search systems.

## Figures and Tables

**Table 1:** Proportion of four types of slopes.

Slope Category	Range (Degree)
Flat land	0–5
Gentle slope	5–10
Steep slope	10–40
Extremely steep	40 or above

**Table 2:** Core characteristics of the three different behavioral strategies.

Behavioral Strategy	Movement Speed	Action Time	Core Characteristic
Remain stationary	0	0	After realizing they are lost, staying put and waiting for rescue is a passive strategy.
Intention to determine the direction of movement	Faster	Shorter	Possesses a sense of direction that is clear or perceived by the self, tending to follow a specific route (e.g., ridges) unless encountering insurmountable obstacles.
No intention to proceed in a specific direction	Slow	Longer	Loss of directional sense, with no fixed direction of movement, easily influenced by terrain and environmental factors, leading to random changes in direction. This is the typical behavior of most missing persons.

**Table 3:** Ablation results for probability guidance and exploration reward.

Algorithm	Overall Success Ratio (%)	Avg. Completion Steps	Success Ratio (Target1) (%)	Success Ratio (Target2) (%)	Success Ratio (Target 3) (%)
Full SPS	88.9	1099.31	94.95	95.66	98.25
No Probability Guidance	63.2	1304.34	83.8	82.6	88.4
No Exploration Reward	41.4	1016.06	74.5	72.2	77
No Both Modules	29	1095.17	62.3	62.5	73.7

## Data Availability

The original contributions presented in this study are included in the article material. Further inquiries can be directed to the corresponding author.

## Appendix A

**Table A1:** Summary of simulation parameters.

Parameter	Value & Description
Number of experiments	2000
Number of targets	3
Maximum steps	2500
DEM source file DEM	ASTER GDEM with 30 m native resolution.
Grid size	50 × 50
Grid spacing	30 m
Search area	1.5 km × 1.5 km
UAV flight height	100 m
Detection radius	30 m
Base speed	1 m/s
UAV speed	10 m/s
Time updating	One global time unit per grid to grid move.
The slope sensitivity coefficient α	7.2
The Probability Recovery Constant λ	1
Replanning interval	25 steps
$\mathrm{Base}\;\mathrm{move}\;\cos t\;\beta_{1}$	0.1
$\mathrm{Slope}\;\mathrm{factor}\;\beta_{2}$	0.5
$\mathrm{Visit}\;\mathrm{penalty}\;\beta_{3}$	0.3
$\mathrm{Local}\;\mathrm{density}\;\mathrm{penalty}\;\beta_{4}$	0.1
$\mathrm{Probability}\;\mathrm{attraction}\;\gamma_{1}$	0.5
$\mathrm{Visit}\;\mathrm{reward}\;\gamma_{2}$	0.5
Random seed	the random number generator is re seeded by the trial index.
